# Beyond boundaries: exploring the role of extracellular vesicles in organ-specific metastasis in solid tumors

**DOI:** 10.3389/fimmu.2025.1593834

**Published:** 2025-06-12

**Authors:** Goutham Nidhi, Vikas Yadav, Tejveer Singh, Deepika Sharma, Monica Bohot, Shakti Ranjan Satapathy

**Affiliations:** 1 Cell and Experimental Pathology, Department of Translational Medicine, Lund University, Skåne University Hospital, Malmö, Sweden; 2 Translational Oncology Laboratory, Department of Zoology, Hansraj College, University of Delhi, New Delhi, India

**Keywords:** extracellular vesicles, metastasis, EMT, cancer, exosome

## Abstract

Extracellular vesicles (EVs) have been identified as important mediators of cancer metastasis, especially in the establishment of organ-specific metastatic niches. These membranous vesicles secreted by tumor cells release diverse bioactive cargo, including proteins, nucleic acids, and lipids, thereby allowing for intercellular communication and microenvironment modulation. Recent evidence demonstrates that EVs can also contribute to the formation of pre-metastatic niches by reprogramming immune cells, modifying the stromal environment, and inducing epithelial-mesenchymal transition (EMT) to promote metastatic colonization. In this review, we describe the molecular mechanism of organotropic metastasis orchestrated by EVs, with special emphasis on immune modulation and tumor microenvironment reprogramming. We also explore the potential of EVs as biomarkers for early detection of metastasis and as potential therapeutic targets for combating metastatic progression. Dissociating EV species and their influence on tumor dissemination will undoubtedly pave the way for implementing novel anti-cancer strategies to intercept tumor dissemination at its very early stages.

## 1 Introduction

Metastasis, the spread of cancer cells from a primary tumor to distant organs, is responsible for the majority of cancer-related deaths (1, 2). Importantly, metastatic dissemination is often organ-specific (organotropic), meaning certain cancers have an affinity to colonize particular organs (1, 3). This concept of “seed and soil,” first proposed over a century ago (4), suggests that disseminating tumor cells (the seeds) can only grow in permissive foreign microenvironments (the soil). However, the molecular mechanisms that prepare a distant organ to become conducive for metastatic growth remained unclear for many years.

**Figure 1:**
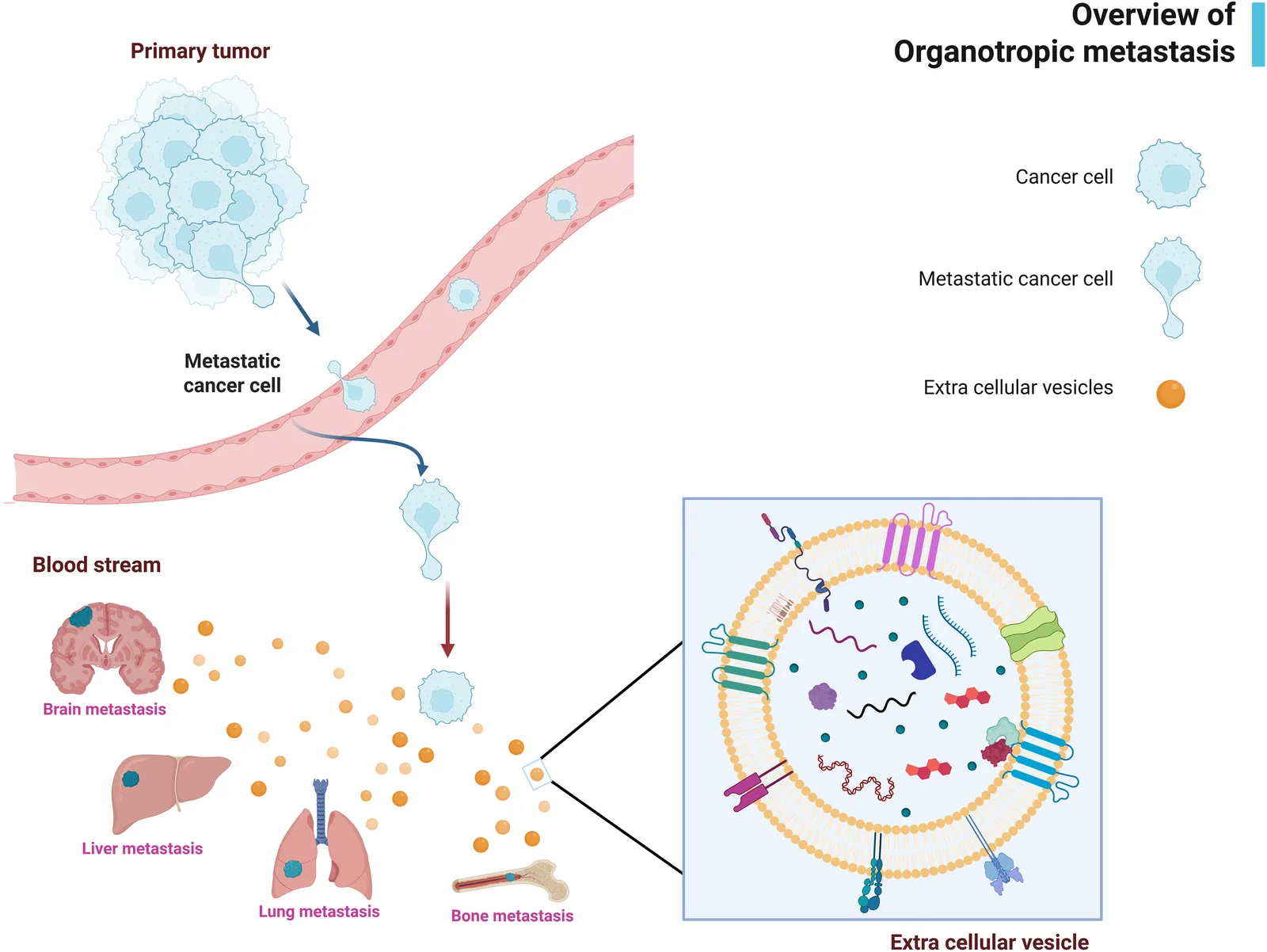
Overview of organotropic metastasis and role of extracellular vesicles (EVs). his schematic illustrates how tumor-derived EVs contribute to organ-specific metastasis. This schematic illustrates how tumor-derived EVs contribute to organ-specific metastasis. The figure highlights the journey of EVs from the primary tumor site to distant organs. Key cargo molecules (e.g., integrins, miRNAs, cytokines) are depicted. Image created with BioRender.com.

Extracellular vesicles (EVs) have recently emerged as critical mediators in preparing this pre-metastatic “soil”. EVs are membrane-bound vesicles released by cells into body fluids, ranging from exosomes of endosomal origin to larger microvesicles shed from the plasma membrane (5, 6). Tumor cells secrete abundant EVs loaded with proteins, nucleic acids, lipids, and other factors that reflect the tumor’s molecular profile. Far from mere cellular debris, these vesicles serve as long-distance communication vehicles that can modulate the behavior of recipient cells and even remodel the microenvironment of distant tissues (7–9). Accumulating evidence indicates that tumor-derived EVs drive organotropic metastasis (3, 10) (**Figure 1**). They can home to specific organs and condition the local milieu to favor subsequent tumor cell colonization. For instance, integrins on the surface of tumor exosomes have been shown to determine their organ specificity, directing exosome uptake by target organ cells and thereby dictating metastatic destination (11, 12). Moreover, EV cargo can reprogram immune cells in target organs, dampening anti-tumor immunity and promoting a tumor-friendly niche. Cancer-derived EVs help “fertilize” distant soils, creating pre-metastatic niches that enable circulating tumor cells to seed and grow successfully (3). This review focuses on the role of EVs in organ-specific metastasis, with particular emphasis on how EV-mediated immune modulation underpins the formation of pre-metastatic niches. We discuss how EVs contribute to each step of the metastatic cascade, from enhancing the invasive capacity of primary tumor cells via epithelial-mesenchymal transition (EMT) to establishing immunosuppressive, pro-metastatic environments in specific distant organs. By examining these processes, we aim to clarify how EVs orchestrate organ-selective metastasis and highlight their potential as targets for novel anti-metastatic therapies.

## 2 Biogenesis of EVs and composition of cargo

EVs have evolved from being considered extracellular debris to recognized mediators of intercellular communication. According to the Minimal Information for Studies of Extracellular Vesicles (MISEV) 2023, EVs are classified into small EVs (<200 nm) and large EVs (>200 nm) (13) and by biogenesis into three main categories: exosomes, microvesicles, and apoptotic bodies. Exosomes (30–150 nm) originate via the endosomal pathway, microvesicles (100–1000 nm) bud directly from the plasma membrane, and apoptotic bodies (500–2000 nm) are released during the final stages of programmed cell death (14, 15). Among these, exosomes have gained particular attention in cancer research due to their ability to carry oncogenic cargo, modulate immunity, and direct organotropic dissemination (10).

The EVs form through two primary membrane budding mechanisms: the endosomal and the plasma membrane pathways (6). The process is well reviewed in a recent report and will not be visually illustrated here (16).

### 2.1 Endosomal pathway in EV formation

EV biogenesis through endosomal pathway begins with the invagination of the plasma membrane to form early endosomes (17). These structures mature into multivesicular bodies (MVBs), which generate intraluminal vesicles (ILVs) via inward budding. Ceramide, a cone-shaped sphingolipid, often facilitates this process. When MVBs fuse with the plasma membrane, ILVs are secreted as exosomes. Alternatively, MVBs may fuse with lysosomes or autophagosomes for degradation (18).

### 2.2 ESCRT-dependent and independent mechanisms of vesicle formation

The formation of ILVs within MVBs is regulated by both endosomal sorting complex required for transport (ESCRT)-dependent and ESCRT-independent pathways (18). The ESCRT machinery consists of four sequentially acting core complexes (ESCRT-0, -I, -II, and -III) and associated regulatory proteins such as the ATPase Vps4. These complexes coordinate the recognition of ubiquitinated cargo and membrane budding into the MVB lumen. In parallel, ESCRT-independent mechanisms, involving lipid molecules like ceramide and proteins such as tetraspanins, also contribute to vesicle formation and cargo selection. Together, these systems ensure the precise biogenesis and secretion of exosomes.

## 3 Relationship between types of EV and molecular cargo

**Figure 2:**
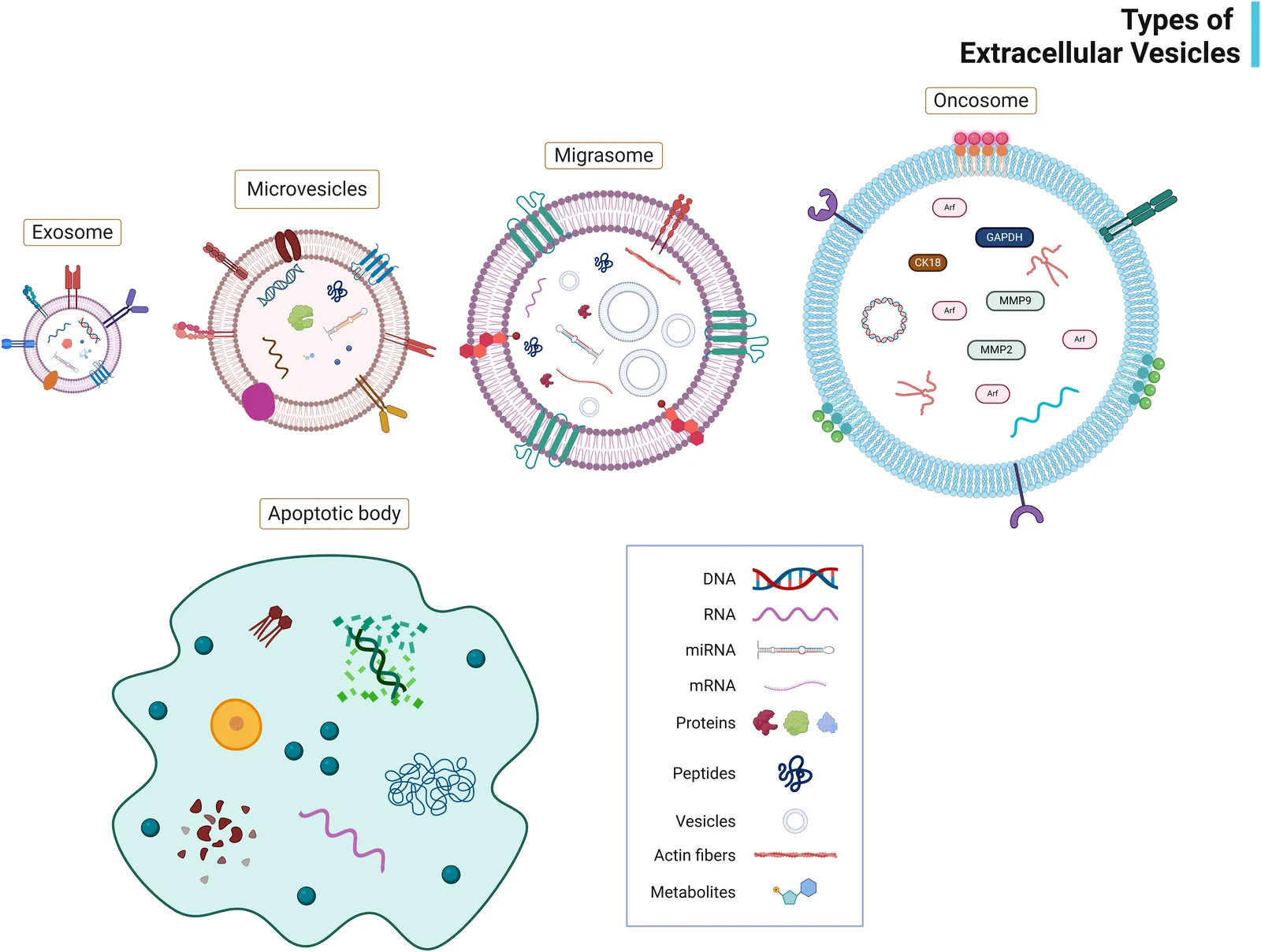
Types of extracellular vesicles (EVs). This figure depicts the major subtypes of EVs, exosomes, microvesicles, migrasomes, oncosomes, and apoptotic bodies classified based on size, consisting of the outer lipid membrane and transmembrane proteins. It illustrates their typical bioactive cargos (proteins, lipids, mRNAs, miRNAs) and surface markers (e.g., CD63, CD81, Annexin V). Image created with BioRender.com.

The molecular composition and biogenesis mechanisms of EVs vary significantly across different vesicle subtypes, each serving distinct biological functions in normal physiology and disease states (19). EVs were often classified as ectosomes (large EVs >200 nm) and exosomes (small EVs <200 nm) based on their size and place of origin (13). Additionally, EVs are classified into many subtypes based on the mode of biogenesis (such as microvesicles, exosomes, autophagic EVs, and apoptosomes) and concept (such as migrasomes, oncosomes, stressome, and matrix vesicles) (20, 21) (**Figure 2****) (****Table 1**). Further refinements in exosome classification have identified distinct subtypes: small exosomes (Exo-S; CD63) (40–80 nm) and large exosomes (Exo-L; CD9) (80–150 nm) (22). Additionally, microvesicles have been categorized into specific subtypes: ARMM (40–100 nm) containing ARRDC1 (arrestin-domain-containing protein-1) and TSG101, whereas regular microvesicles (150–1000 nm) and oncosomes (1-10 µm) contain annexin A1 (23).

**Table 1:** Classification of extracellular vesicles.

Name	Category	EV class	Size	Biogenesis	Markers
Exosome	Microvesicles	Small EV	30–150 nm	Multivesicular endosome	CD63, CD9, CD81, TSG101, Alix
Microvesicles	Microvesicles	Large EV	100–1000 nm	Plasma membrane shedding	Annexin A1, ARF6
Large oncosome	Microvesicles	Large EV	1-10 μm	Plasma membrane shedding	Annexin A1, ARF6
ARMM	Microvesicles	Small EV	40–100 nm	Plasma membrane shedding	ARRDC1, TSG101
Migrasome	Migrasome	Large EV	500–3000 nm	Migracytosis	TSPN4, TSPN6, Integrins
Apoptotic bodies	Apoptotic EV	Large EV	50–2000 nm	Apoptosis	Annexin V, PS
Autophagic EV	Autophagic EV	Small to large EV	40–1000 nm	Autophagosome endosome fusion (Amphisome)	LC3B-PE, p62 dsDNA/Histones
Stressome	Stressed EV	Small to large EV	40–1000 nm	Plasma membrane shedding, autophagy	HSP90, HSPs
Matrix vesicles	Matrix vesicles	Small to large EV	40–1000 nm	Matrix binding and release	Fibronectin, proteoglycans

### 3.1 Microvesicles

Microvesicles, also termed ectosomes, form through the direct outward budding of the plasma membranes and transport a diverse cargo of bioactive molecules. This cargo includes epithelial growth factor receptors (EGFR) that mediate cell proliferation signals, matrix metalloproteinases (MMPs) that facilitate extracellular matrix (ECM) remodeling, and vascular endothelial growth factors (VEGF) that promote angiogenesis (24). The formation of microvesicles begins with the activation of Small GTPase proteins that initiate plasma membrane budding. This is often assisted by floppies that facilitate the translocation of phosphatidylserine from the inner to the outer leaflet of the plasma membrane.

The ATP-binding cassette transporter 1 (ABCA1) plays a crucial role by promoting asymmetric phospholipid distribution, creating structural imbalance within the plasma membrane. This imbalance triggers extracellular signal-regulated kinase (ERK) activation, leading to myosin light chain kinase (MLCK) phosphorylation and ultimately resulting in the scission of ectosomes from the plasma membrane. The ESCRT machinery, mainly through the ALG-2-interacting protein X (Alix) interaction with the ESCRT-III complex, facilitates exosome biogenesis and ectosome secretion into the ECM.

### 3.2 Migrasomes

Migrasomes represent a recently identified class of EVs formed when retraction fibers are released from the trailing edge of migrating cells (25). These distinctive structures range from 500–3000 nm in diameter and display a characteristic pomegranate-like morphology containing multiple smaller vesicles in their lumen. While sharing some morphological features with MVBs, migrasomes notably lack the lysosomal-associated membrane protein 1 (LAMP1), a typical MVB marker. Their biogenesis depends on three key processes: actin filament formation, enrichment of integrin adhesion receptors, and generation of tetraspanin-rich microdomains, particularly tetraspanin-4 (TSPN4). Migrasomes are enriched with proteins implicated in cell migration, tumor invasiveness, cell adhesion, and cell-ECM interactions, suggesting their importance in tissue remodeling and cancer progression (26).

### 3.3 Apoptotic bodies

Apoptotic bodies are released from membrane blebs during the controlled process of programmed cell death (27). The cargo of apoptotic bodies varies based on their origin, size, and cause of apoptosis (28). Apoptotic bodies contain 1028 proteins (annexin, RAB11, cytosolic, heat shock proteins), DNA, microRNA, and lipids. Their formation is initiated by CASPASE-3 cleavage of Rho-associated protein kinase-1 (ROCK1), which induces phosphorylation of the MLC and subsequent contraction of cortical actin-myosin networks (29). During apoptosis, phospholipid scramblase proteins such as transmembrane protein 16F (TMEM16F) and XK-related protein 8 (Xkr8) facilitate the exposure of phosphatidylserine on the outer leaflet of the plasma membrane. This externalized phosphatidylserine interacts with bridging molecules like Annexin V, milk fat globule-EGF factor 8 (MFG-E8), and growth arrest-specific protein 6 (Gas6), effectively marking these vesicles for recognition and clearance by phagocytic cells (30, 31).

### 3.4 Cancer-specific EVs

Oncosomes, a cancer-specific EV subtype, carry molecular cargo that promotes tumor metastasis. This includes specific coding and noncoding RNAs, microRNAs (miRNAs) (e.g., miR-1227 and miR-125a), the membrane protein caveolin-1, matrix-degrading enzymes such as MMPs, and ADP-ribosylation factor 6 (ARF6) (32, 33). These vesicles are enriched with enzymes involved in tumor-associated metabolic pathways and are typically formed by the shedding of non-apoptotic membrane blebs from amoeboid-migrating cancer cells.

In addition to conventional transmembrane and cytosolic proteins, EVs, including exosomes, oncosomes, and blebbisomes, also incorporate glycosylphosphatidylinositol (GPI)-anchored proteins (GPI-APs) (34, 35). These lipid-linked proteins play essential roles in immune modulation, cell adhesion, and signal transduction. Their selective enrichment in certain EV subtypes suggests functional specialization. For instance, GPI-APs such as uPAR (urokinase-type plasminogen activator receptor) and Thy-1 have been detected in large EVs like oncosomes and blebbisomes, contributing to tumor invasion and cell motility (21). The presence of these GPI-APs may influence EV uptake, immune evasion, and pre-metastatic niche remodeling during the metastatic cascade. However, their precise mechanistic roles remain an area of active research. A recent review has elegantly explored the biogenesis and functional relevance of GPI-APs in tumor-derived EVs using colorectal cancer (CRC) as an example; hence, it will not be covered here (36).

Tumor-derived EVs have emerged as key regulators of organotropic metastasis, capable of pre-conditioning target tissues into pre-metastatic niches in specific organs. For example, integrins on exosomes (such as α6β4 and αvβ5) drive organ-specific metastasis to the lung and liver, respectively, by directing exosome uptake into resident organ cells and activating pro-metastatic signaling (11).

A recently discovered class of exceptionally large EVs, termed ‘blebbisomes’ (~ 20 μm in diameter), contains intact cellular organelles such as mitochondria, despite lacking a nucleus (37). These cell-sized vesicles are shed by aggressive cancer cells and have been detected in distant bone marrow. Notably, blebbisomes are enriched with immune checkpoint proteins, including programmed death-ligand 1 (PD-L1), PD-L2, B7 homolog 3 (B7-H3), and V-domain Ig suppressor of T cell activation (VISTA), consistent with the report of Chen et al. (38). This immunosuppressive cargo suggests that tumor-derived EVs provide organ-specific “zip codes” directing metastasis and actively suppress local immunity to establish a permissive microenvironment in target organs.

### 3.5 Exosomes

Exosomes are the most studied EVs developed by the inward budding of endosomes. The intraluminal vesicles (ILVs) found in the multivesicular bodies undergo exocytosis, or fusion with the plasma membrane, and are released as exosomes into the extracellular matrix (24). The ectosomes (shedding microvesicles) are released by the outward budding of the plasma membrane (ectocytosis). In contrast, the apoptotic cells release apoptotic bodies via plasma membrane blebbing (39). The biogenesis mechanisms of these three EVs are antagonistic, whereas those of other types are more conceptual. A lipid bilayer membrane is present in the EVs, which protects the contents during intercellular transport, and its composition is different from that of the donor cell (40).

Exosomes contain proteins (Alix, TSG101, HSP70, integrins), lipids (cholesterol, ceramide, sphingolipids), glycan, polysaccharides, mRNA, miRNA, and are enriched by CD63, CD9, and CD81. Exosomes originate from cancer cells with DNA, RNA, and mutated proteins. The cargo of ectosomes is almost like exosomes and contains MMPs (e.g., MMP2), cytoskeletal proteins (e.g., α-actin and β-actin), integrins, ribosomal proteins, mitochondrial proteins, and centrosomal proteins.

## 4 Regulatory mechanisms of EV formation and release

Tetraspanins (TSPNs) play a pivotal role in EV biogenesis and function. These small integral membrane proteins, characterized by four transmembrane domains, include 33 different variants identified in humans (41). Different TSPN proteins serve distinct functions in vesicle formation. High expression of TSPN6 promotes exosome release, while cluster of differentiation 81 (CD81) and CD82 regulate the formation of plasma membrane-derived EVs and influence membrane morphogenesis. Tetraspanins may also modulate actin cytoskeleton reorganization through interaction with Rho family GTPases, potentially influencing cell motility and invasion capacity (42, 43). Studies in prostate cancer cells showed that CD9 knockdown and CD151 overexpression altered the EV proteome composition, enhancing their migratory and invasive characteristics. This demonstrates how cargo alterations in EVs regulated by TSPNs can significantly impact cellular migratory and invasive features (44).

Exosome secretion is a highly regulated process that involves the release of vesicles, often of endocytic origin, into the extracellular environment. During this phase, MVBs produced by endocytosis either fuse with lysosomes for destruction or with the plasma membrane for exosome release (45). A number of components, including SNARE proteins, microtubules, the actin cytoskeleton, and Rab GTPases, work together to orchestrate this secretion efficiently. The end stage of exosome release involves SNARE-mediated membrane fusion, where v-SNAREs on the MVB membrane interact with t-SNAREs (such as SNAP23) on the cell membrane. Phosphorylation of SNAP23 promotes the formation of the SNARE complex, which in turn mediates exosome secretion (45).

The precise compositions of the diverse cargo found in EVs, including exosomes, vary based on several factors, such as the kind of cell, the manner of biogenesis, and physiological conditions. The cargo mostly comprises proteins, lipids, and nucleic acids. Common proteins in EVs are either associated with the biogenesis mechanisms, signal transduction, and antigen presentation, or are transmembrane proteins (45, 46). Specific lipids, including sphingomyelin, cholesterol, desaturated lipids, phosphatidylserine, and ceramide, are linked to distinct EV types, and the lipid composition of EVs is similar to that of the cells of origin. MVB-derived EVs have more phosphatidylserine, which helps recipient cells internalize them, even though EV lipids typically lack phosphatidylcholine and diacylglycerol (45, 46).

The genetic content of EVs is diverse, encompassing DNA and various RNA types, with a particular enrichment with small RNAs up to 4 kb in size. These RNA molecules can exist in different forms: associated with ribonucleoproteins like argonaute 2 (AGO2), bound to high-density and low-density lipoproteins (HDLs and LDLs), or directly connected to EVs. The precise detection of RNA distribution among these carriers depends on the isolation methods employed (47, 48).

## 5 Function of EVs in intercellular communication

Communication between EVs and recipient cells primarily occurs through the horizontal transfer of cargo, particularly mRNA and miRNA, along with other bioactive molecules. EVs have surface molecules that enable attachment to the recipient cells and initiate signal transduction to modulate the functional properties of the recipient cell. EVs generated from malignant cells contain bioactive materials with oncogenic properties, and their DNA could serve as diagnostic biomarkers (49). These EVs play a significant role in mediating communication between malignant cells and tumor-associated cells.

## 6 Significance of understanding EVs in relation to metastasis

Most cancer-related fatalities are caused by metastasis (95%) (50). EVs, especially exosomes, play a critical role in EMT, a key driver of metastasis (51, 52). A detailed account of EMT and the signaling pathways involved is presented in the dedicated section “EVs and EMT.” The mutated nucleic acids and oncogenic proteins in the EVs act on recipient cells, leading to tumorigenesis, metastasis, and drug resistance (53, 54). Exosomes have been identified to promote the growth and progression of various cancers, including breast, prostate, lung, and pancreatic cancer (55).

Cancer cells alone cannot mediate metastasis; a collective effort must be made to support the tumor environment. Only a few proportions of primary tumors can form micrometastatic foci in nonmalignant tissues via several pathways (56). These metastatic niches occur through cancer invasion into the basement membrane and extravasation into the bloodstream. The tumor cells, during metastasis, pass through harsh environments (blood and lymph shear stress), cross endothelial barriers, evade immune surveillance, proliferate, and finally adapt to the microenvironment (57, 58). Tumor-derived EVs contribute to pre-metastatic niche formation by reprogramming immune and stromal cells. The immunological and molecular processes driving this phenomenon are discussed in the “EVs in Pre-Metastatic Niche Formation and Immune Modulation” section.

Before cancer cells metastasize, epithelial cells show mesenchymal characteristics (increased motility and reduced adhesion), enriching the tumor cells with metastatic properties (59). Thus, the EMT enables the migration of carcinoma cells to distant organs, regulated by miRNAs and various pathways (51). EVs are long-lived signaling molecules with a high degree of selectivity in the circulatory system (60). This EV characteristic is used by the tumor cell to locate target tissues and create metastatic niches (61). EVs recruit mesenchymal stem cells (MSCs), which influence metastasis within the tumor microenvironment (TME) and play a crucial role in MSC-mediated metastasis (62).

It was recently found that EVs control the intracellular metabolism of tumors and the availability of nutrients in the TME, which encourages metastasis (63, 64). EV cargo, including proteins and miRNAs, plays critical roles in modulating the TME, driving angiogenesis, and initiating EMT. Specific signaling pathways influenced by EVs, such as TGF-β, WNT/β-catenin, and PI3K/AKT, are explored in detail later in this review (65). The release of EVs from cancer cells causes metastasis in TME under extreme conditions (nutrient deprivation, acidity, and hypoxia); even nonmalignant cells leak EVs that affect metastasis in TME (66, 67).

### 6.1 EVs and the TME

Notably, EVs, particularly small EVs (sEVs), significantly impact numerous stages of the metastatic cascade, contributing to the spread of cancer (8). Tumor-derived sEVs directly influence the motility and invasiveness of tumor cells by induction of directional motility via ECM components and the facilitation of invasive structures, such as invadopodia (68–70). EVs can contribute to the degradation of the ECM by spreading MMPs present both in small EVs and large EVs shed by tumors. The sEVs from cancer-associated fibroblasts (CAFs) may also trigger an invasive response in recipient cancer cells due to the activation of some signaling pathways. Tumor sEVs reprogram the physiology of neighboring and distant non-tumor cells to support the spread and growth of disseminated cancer cells, mainly through the induction of vascular permeability and conditioning pre-metastatic niches in target organs (71). They could do this by interacting with specific target cells that will subsequently activate relevant signaling pathways to form pre-metastatic niches in remote organs. It is unclear how sEVs, which are released from the primary tumor body, function *in vivo* to encourage the development of this pre-metastatic niche.

In contrast to the biogenesis of sEVs, a recent example is the knockdown of RAB proteins such as RAB27A, which reduces the secretion of sEVs and inhibits metastasis in animal models (72). However, targeting such molecules, like RAB27A, also affects other cellular processes and secretions. Thus, complementary methodologies need to be developed to understand the functions of EVs *in vivo*. Proteoglycans (PGs) are such macromolecules consisting of a core protein decorated with chains of glycosaminoglycans, such as heparan sulfate (HS) and chondroitin sulfate, which are highly polyanionic due to sulfation, and thus determine their interactions with various ligands. PGs are known to form a crucial part of the ECM in mammalian tissues and participate in different pathophysiological processes. It has been demonstrated that HSPGs sequester and bind pro-tumorigenic factors like growth factors, cytokines, and chemokines that aid in tumor progression. HSPGs are once again at the core of EV-mediated intercellular communication, as recent studies have identified them for both exosome synthesis and EV uptake (73–75). PG remodeling encompasses changes in glycosaminoglycan content and structure and the altered expression of core proteins, all factors highly important in understanding the principle behind EV-mediated cell-to-cell signaling. Membrane PGs are crucial players in EV formation and function and play roles in EV biology and intercellular communication.

Proteins, mRNAs, miRNAs, and other noncoding RNAs can all be loaded into EVs, and research into the transfer of miRNAs through EVs is ongoing, particularly in oncology. Functional transfer of EV-mediated miRNAs is rarely clearly proved, despite the general agreement that EV-transferred miRNAs can alter recipient cells’ gene expression. The molecular conformations by which EVs mediate the transfer of miRNAs into recipient cells, including how miRNAs associate with RISC in recipient cells, are still poorly understood (76–78). Some publications indicate that pre-miRNAs loaded into the RISC machinery could be secreted by tumor cells in EVs and might undergo miRNA maturation extracellularly (79). Indeed, the exact nature of the carrier of RISC/miRNA in EVs and the relevance of EV-mediated miRNA transfer in cancer, including *in vivo*, has not yet been proven. Tosar and Cayota have extensively reviewed how tRNA fragments encapsulated in sperm regulate gene expression in embryos (80). These findings point out the role of EVs in transporting regulatory RNAs from generation to generation.

### 6.2 Interactions between EVs and immune cells

The TME comprises various immune cells, including macrophages, dendritic cells, lymphocytes, neutrophils, myeloid-derived suppressor cells, and natural killer cells, all influencing tumor progression (81). Tumor-associated macrophages (TAMs) are the most common among these cell types and communicate with tumor cells in both directions through EVs, encouraging metastasis (82). Tumor-derived EVs can induce macrophage polarization toward the M2 phenotype, enhancing tumor cell motility, invasion, and EMT (83). Cal et al., in their study, showed that EVs containing THBS1 polarize macrophages towards an M1 phenotype in oral squamous cell carcinoma cells, but those expressing miR-29a-3p and CMTM6 cause macrophages to shift towards an M2 phenotype, which contributes towards metastasis (84). Similarly, Wang and Qiu, reported that EVs enriched with miR-301a promote M2 polarization through the PTEN/PI3Kγ pathway in pancreatic cancer, which increases their motility and invasion (85). EVs produced from CRC have miRNAs such as miR-25-3p, miR-130b-3p, and miR-425-5p, stimulating M2 polarization via the PTEN/PI3K pathway (86). This process enhances the EMT, increases VEGF secretion, and fosters tumor cell escape (87). Likewise, EVs from liposarcoma contain miR-25-3p and miR-92a-3p, which stimulate interleukin 6 (IL-6) secretion by macrophages, ultimately increasing tumor cell invasiveness (88).

M2 TAMs can further influence tumor progression by secreting EVs that modulate tumor cells. M2-derived EVs enriched with miR-155 and miR-196a-5p inhibit the tumor-suppressor gene RASSF4 in non-small cell lung cancer, encouraging their invasiveness (89). Pancreatic adenocarcinoma-derived M2 EVs contain miRNA-501-3p, which activates TGF-β signaling, causing enhanced invasiveness (90). Similarly, in esophageal cancer, EVs carrying long noncoding RNA (lncRNA) downregulate miR-26a in tumor cells, upregulating ATF2 and promoting metastasis (91). In gastric cancer, M2 TAM-derived EVs transport ApoE, which activates PTEN/PI3K signaling and remodels the cytoskeleton to facilitate migration (92).

Tumor-associated neutrophils, particularly N2 neutrophils, also contribute to metastasis by aiding pre-metastatic niche development, promoting angiogenesis, and assisting tumor cells in extravasation (93). Emerging research suggests tumor-derived EVs may activate neutrophils in pre-metastatic niches through pathways such as toll-like receptor 3 (TLR3) signaling in lung metastasis or NF-κB induction in gastric cancer. However, further investigation is required to fully elucidate the function of EVs in neutrophil-driven metastasis (94).

The initial phase of the pre-metastatic niche formation involves macrophage recruitment, driven by EV-mediated signaling from tumor cells. In pancreatic cancer, EVs containing macrophage inhibitory factor (MIF) selectively interact with Kupffer cells in the liver, leading to TGF-β secretion. Hepatic stellate cells are then activated, generating fibronectin and enlisting bone marrow-derived macrophages to prime the niche (95). Similarly, the lungs are also home to EVs originating from breast cancer that include ANXA6, which is released in response to chemotherapy. These EVs activate the CCL2-CCR signaling axis, drawing monocytes that mature into macrophages at metastatic locations (96).

Once recruited, macrophages become polarized in reaction to EVs produced from cancer, creating an environment that supports tumor growth. In ovarian cancer, EVs carrying miR-21-3p reach the pre-metastatic niche, activating the STAT3 pathway and promoting M2 macrophage polarization (97). This process results in immune suppression and increased IL-6 secretion, further reinforcing STAT3 signaling. A similar mechanism occurs in CRC, where tumor-derived EVs enriched with miR-21-5p interact with TLR7 on Kupffer cells in the liver, driving macrophage polarization and IL-6 secretion. Understanding these EV-mediated mechanisms could provide valuable insight into therapeutic strategies to modulate immune responses in metastatic cancers (98).

## 7 EVs drive the metastatic cascade

Once tumor cells acquire invasive capabilities, EVs act as important facilitators at multiple steps of the metastatic cascade. One key step is the EMT, wherein carcinoma cells shed epithelial traits and gain mesenchymal, migratory properties necessary for dissemination. The mechanisms by which EVs promote EMT are detailed in the section “EVs and EMT.” Beyond EMT induction, EVs contribute to other early metastatic events. They can promote localized invasion by remodeling the ECM. Tumor EVs often contain matrix-degrading enzymes (e.g., MMPs) that facilitate ECM degradation when delivered to neighboring stromal cells or directly deposited into the matrix (99, 100). In addition, EVs stimulate the formation of invasive structures; for instance, small EVs from CAFs have been reported to enhance invadopodia formation in cancer cells, aiding tissue penetration (101). EV cargo, such as chemokines and integrins, can also increase tumor cell motility and guide directional migration toward blood vessels. Simultaneously, EVs can carry immunomodulatory molecules that aid metastatic cells in evading immune surveillance during transit. For example, some EVs carry programmed death-ligand 1 (PD-L1) on their surface, which can bind and inhibit T cells, thereby protecting circulating tumor cells from immune attack (38). Thus, tumor-derived EVs significantly amplify metastatic efficiency from the primary site through a combination of biochemical and immune-modulating effects. However, the influence of EVs is perhaps most profound in their ability to prepare future metastatic sites. Rather than metastasis occurring in purely receptive organs by chance, tumor EVs actively condition specific distant organs even before cancer cells arrive. This pre-conditioning involves establishing a hospitable microenvironment known as the pre-metastatic niche. The organ-specific nature of this process is remarkable; EVs seem to “know” where to go and what changes to induce upon arrival. The following section discusses how EVs modulate barrier function and cytoskeletal dynamics, home to particular organs, and orchestrate pre-metastatic niche formation, mainly by recruiting and reprogramming immune cells in those target tissues for an efficient metastasis.

### 7.1 EVs in modulating barrier function and cytoskeletal dynamics

EVs play a crucial role in modulating epithelial and endothelial barrier integrity, a key step in metastasis (11, 102). Tumor-derived EVs have been shown to disrupt tight junctions and increase vascular permeability, thereby facilitating tumor cell intravasation and extravasation (11). For instance, EVs enriched with VEGF, TGF-β, or MMPs can compromise endothelial barrier function by degrading junctional proteins like claudin-5, occludin, and ZO-1, promoting paracellular permeability at distant metastatic sites (102). In melanoma, cancer cells secreted EVs promote vascular permeability by upregulating inflammatory mediators such as S100A8, S100A9, and TNF-α, leading to bone marrow progenitor cell recruitment (103). Similarly, exosomes derived from human breast cancer cells induce vascular leakiness in the lung through S100 protein upregulation and Src kinase activation, highlighting a mechanism of organ-specific endothelial priming (11). Additionally, metastatic breast cancer cells release miR-105-enriched exosomes that directly target and degrade tight junction protein ZO-1 in recipient endothelial cells, compromising barrier integrity and increasing susceptibility to metastatic invasion (104). Collectively, these findings suggest that tumor-derived EVs mediate endothelial barrier disruption; however, further studies are needed to delineate the organ-specific mechanisms by which EVs regulate vascular integrity.

Moreover, EVs contribute to cytoskeletal remodeling in both tumor and stromal cells. By delivering active molecules such as Rho GTPases, integrins, tetraspanins (e.g., CD9, CD81), and integrins, EVs induce actin cytoskeleton reorganization, which enhances cell motility, invasion, and the formation of invasive structures like invadopodia (44, 100, 101). This cytoskeletal reprogramming facilitates EMT and primes stromal and endothelial cells in the pre-metastatic niche to adopt pro-invasive phenotypes. EVs from CAFs and hypoxic tumor cells have been reported to influence the expression and activity of actin-binding proteins (e.g., cofilin, fascin) and promote membrane ruffling, lamellipodia, and filopodia formation in recipient cells (68). The cytoskeletal alterations are crucial for tumor cell migration and successful colonization at secondary sites. Taken together, EV-mediated barrier disruption and cytoskeletal reorganization are central to tumor progression and represent additional layers of complexity in the metastatic cascade.

### 7.2 EVs function in fostering a conducive environment for metastatic colonization

EVs are crucial in forming pre-metastatic niches, essential for colonizing distant organs by metastatic cancer cells. *Lyden* proposed the idea of a pre-metastatic niche (105). The pre-metastatic niche is characterized by four stages, as stated by Liu and Cao: tumor-derived secreted factors (TDSFs), Bone marrow-derived cells (BMDCs), suppressive immune cells, and host stromal cells (106). *Hoshino* discovered that exosomes produced from tumors played a decisive role in organ-specific metastasis (11). Exosomes from tumors mediate non-random transfer patterns by creating a favorable microenvironment at potential metastatic sites. Exosomes play a crucial role in metastasis by actively homing to metastatic sites, influencing the spread of cancer cells, and redirecting their migration. Their organ-specific targeting is driven by surface integrins, allowing selective uptake by recipient cells and ultimately facilitating metastatic progression (107).

Tumor development and metastasis largely depend on chronic inflammation, creating a pre-metastatic niche in the local inflammatory milieu. EVs can upregulate pro-inflammatory genes, recruit immune cells, and create a supportive environment for tumor growth. The biological cargo carried by EVs can trigger modifications that support a pre-metastatic niche, like improving angiogenesis and enabling long-distance cellular communication (108). Moreover, leaky blood vessels help create a pre-metastatic niche by attracting circulating EVs (109). The cancer-derived exosomes inherit the organotropism of their parent cancer cell, which targets niche cells at various metastatic locations (11). Oncoprotein MET (found in metastatic melanomas) instructs bone marrow progenitor cells to adopt a vasculogenic phenotype to form the pre-metastatic niche in the lungs (103).

### 7.3 EVs and EMT

EVs are crucial in promoting EMT in cancer, which is characterized by a lack of epithelial polarity and cell-cell adhesion, whereby the epithelium transforms into mesenchymal-like cells with increased motility, enhancing the metastatic propensity of malignant cells. EVs orchestrate EMT by transferring biological molecules (e.g., proteins, lipids, and nucleic acids), between cancer cells and the TME, including mRNAs, miRNAs, and lncRNAs. Indeed, CAFs or hypoxic tumor cells secreted EVs, have been reported to carry EMT-driving molecules, such as TGF-β and HIF-1α, and specific miRNAs, such as miR-21 and the miR-200 family (51). The uptake of these EVs by less aggressive cancer cells can trigger the downregulation of E-cadherin and the upregulation of mesenchymal markers like vimentin, thereby increasing the motility and invasiveness of tumor cells. EV-associated miRNAs (such as members of the miR-200 family or miR-21) can also silence epithelial maintenance genes in target cells, further driving EMT and metastatic potential.

### 7.4 Signaling pathways involved in EV-mediated EMT

EVs induce several major signaling pathways to mediate the EMT of cancer cells.

#### 7.4.1 TGF-β signaling

EVs have been shown to transport a wide range of bioactive materials, including proteins, mRNAs, and noncoding RNAs connected to TGF-β signaling. These elements can alter recipient cell activity, affecting metastasis and carcinogenesis. TGF-β is known to induce EMT, enhancing the invasiveness and metastatic potential of cancer cells (110–112). EVs can migrate the active TGF-β receptors from cancerous cells to surrounding cells and stimulate the TGF-β signaling in recipient cells (113). TGF-β signaling is initiated when ligands bind to type I and II receptors, inducing their oligomerization and activating protein kinase activity. The best-studied co-receptor for TGF-β is the type III receptor that binds all three TGF-β isoforms with high affinity and presents them to the signaling complex that further recruits signaling proteins (113). Upon phosphorylation, SMAD (the substrate of TGFβRII) oligomerizes with SMAD4, enters the nucleus, and regulates gene transcription. This pathway is also greatly influenced by non-protein-coding RNAs, such as miRNAs and lncRNAs (114, 115).

#### 7.4.2 WNT/β-catenin pathway

In the TME, the WNT/β-catenin pathway is essential, especially when EVs are involved. WNT signaling is crucial for several cellular processes, including cell migration, differentiation, and proliferation, and its dysregulation is commonly linked to the development of cancer (116). Mutant versions of β-catenin, frequently present in various malignancies, especially CRC, can be carried by EVs. When recipient cells have wild-type β-catenin, these mutant β-catenin can trigger WNT signaling. By encouraging the transcription of WNT target genes important in cell proliferation and survival, this mechanism accelerates the growth and progression of tumors (117). WNT ligands such as WNT3A and WNT5A are transported between cells by EVs. Depending on the situation and the kind of receptors found in recipient cells, these ligands can either stimulate or inhibit WNT signaling pathways (117). EVs activate the WNT/β-catenin signaling pathway, causing stabilization and nuclear translocation of β-catenin. The latter is required for transcription genes responsible for EMT, such as Snail and Twist.

#### 7.4.3 PI3K/AKT and MAPK/ERK pathways

PI3K/AKT and MAPK/ERK pathways are crucial signaling cascades that significantly influence TME by interacting with EVs (32). These pathways play a role in growth, survival, and metastasis, among other cellular functions, and cancer progression is frequently linked to their dysregulation. Phosphorylated AKT and other active elements of the PI3K/AKT signaling pathway can be carried by EVs. These EVs can activate PI3K/AKT signaling when they are absorbed by recipient cells, which improves cell survival and proliferation. In this aspect, Liem et al. have shown that insulin therapy increases the amount of EVs secreted by CRC cells, which are loaded with carcinogenic cargo that encourages the formation of tumors (118). EVs may transfer oncogenic proteins and miRNAs that activate PI3K/AKT and MAPK/ERK signaling cascades. These pathways support EMT by promoting cell survival, migration, and invasion while repressing epithelial characteristics.

#### 7.4.4 Notch signaling

Notch signaling is well-known for determining the fate of cancer cells, promoting their growth, and preserving their stem-like characteristics. EVs can carry ligands for the Notch receptor, activating Notch signaling in recipient cells. Small EVs from cancer cells can package and transfer notch signaling components, like the Notch intracellular domain (NICD). EVs and Notch signaling can interact through non-classical routes that bypass conventional ligand-receptor interactions (119). By regulating Notch activity more sophisticatedly, this pathway enables tumor cells to engage with the TME and modify their behavior efficiently. For example, without direct contact between donor and recipient cells, small EVs can activate Notch signaling, thereby increasing the aggressiveness of tumors (119). The EMT process, essential for cancer invasion and metastasis, is intimately related to Notch signaling. When this pathway is dysregulated, cancer cells may exhibit EMT traits that improve their capacity for migration (120).

## 8 EVs and pre-metastatic niche formation

### 8.1 EVs in pre-metastatic niche formation and immune modulation

A pre-metastatic niche is a favorable microenvironment established in a distant organ prior to the arrival of CTCs. Tumor-derived EVs are now recognized as key instigators of pre-metastatic niche formation, largely by mobilizing and reprogramming immune cells in target organs (121). One well-characterized example is pancreatic cancer, which preferentially metastasizes to the liver. Pancreatic tumor exosomes carrying MIF home to the liver and specifically interact with Kupffer cells (resident hepatic macrophages), triggering the release of TGF-β (95). The increase in TGF-β activates hepatic stellate cells to produce fibronectin, a matrix protein that helps recruit bone marrow-derived monocytes to the liver. These monocytes then differentiate into macrophages within the nascent niche, completing an EV-driven loop of immune cell recruitment and activation that primes the liver for metastasis (95). Similarly, in breast cancer, chemotherapy stress can stimulate tumor cells to shed EVs enriched in annexin A6 (ANXA6). These ANXA6^+^ EVs travel to the lung and induce resident lung cells to secrete C-C motif chemokine ligand 2 (CCL2), attracting CCR2^+^ monocytes into the pre-metastatic niche. The recruited monocytes mature into pro-tumoral macrophages at the metastatic site (96). These examples illustrate how tumor EVs lay the groundwork by orchestrating the influx and localization of myeloid cells in a specific organ. Once immune cells have been recruited to a future metastatic site by EV signals, tumor-derived EVs continue to modulate their phenotype toward a pro-metastatic, immunosuppressive state. In the pre-metastatic niche, arriving macrophages are often skewed toward an alternatively activated, M2-like phenotype that promotes tumor growth. EV cargo plays a direct role in this polarization. For instance, ovarian cancer-derived EVs carrying miR-21-3p have been found to enter resident macrophages at distant sites and activate the STAT3 signaling pathway, driving these macrophages into an immunosuppressive M2 state (97). These EV-educated M2 macrophages secrete IL-6 and other factors that further reinforce STAT3 activation in a positive feedback loop while suppressing local anti-tumor immune responses. In CRC, tumor EVs enriched with miR-21-5p similarly engage TLR7 on liver Kupffer cells, inducing them to produce IL-6 and adopt an M2 polarization, thereby establishing an inflammatory, tumor-promoting niche in the liver (98). The immunosuppressive milieu is compounded by the expansion of regulatory T cells and myeloid-derived suppressor cells (MDSCs) that are often drawn into or activated within the niche, partly in response to EV-induced cytokines and chemokines. In addition to macrophages, other immune and stromal components are influenced by EVs during niche formation. Neutrophils, for example, can be activated by tumor EVs in pre-metastatic sites. Studies suggest that EV-associated “danger signals” (such as specific RNAs or heat shock proteins) engage pattern recognition receptors on neutrophils, leading to a pro-inflammatory neutrophil response that paradoxically supports metastasis (122). These activated neutrophils (sometimes termed N2 neutrophils) secrete factors that enhance tumor cell extravasation and seeding and promote angiogenesis in the pre-metastatic organ. Likewise, EVs may directly condition other stromal cells, such as fibroblasts and endothelial cells in the target organ. For instance, EV uptake can prompt local fibroblasts to become pro-inflammatory and pro-fibrotic or cause endothelial cells to upregulate adhesion molecules that increase vascular permeability and cell adhesion. Such changes in the stroma make the tissue more amenable to subsequent cancer cell invasion. Crucially, tumor EVs often carry oncoproteins and immunomodulatory molecules, ensuring any arriving cancer cells will face reduced immune resistance. Tumor-derived EVs have been found to contain immune checkpoint proteins such as PD-L1 and other suppressive ligands (37). By depositing these factors into the pre-metastatic organ, EVs create localized immunosuppression; resident T cells, natural killer cells, and other immune effectors are functionally inhibited even before tumor cells arrive. This means that when cancer cells finally appear, they encounter a “primed” microenvironment replete with supportive stromal cells, growth factors, new vasculature, and subdued immune surveillance. Altogether, the actions of EVs ensure that the pre-metastatic niche is rich in growth-permissive signals (e.g., fibronectin, S100 proteins, VEGF), pro-inflammatory mediators (e.g., TNF-α, IL-6) that paradoxically aid tumor development, and immunosuppressive cell populations. In essence, EVs rewire the normal tissue homeostasis of target organs into a pro-metastatic configuration. Through these concerted effects, EVs impart organotropism to metastasis. The specificity of EV targeting is partly dictated by molecules on their surface (certain integrin combinations on EV membranes can direct them preferentially to lungs vs. liver, for example) (11). Once docked in the target organ, EVs unleash a cascade of molecular events, recruit bone marrow progenitors, educate macrophages and neutrophils, alter the vasculature, and suppress adaptive immunity that establishes a niche conducive to metastatic colonization. This multi-pronged remodeling of distant tissues by tumor EVs is a driving force behind organ-specific metastasis, highlighting that metastasis is not solely a property of the cancer cell (“seed”) but also a result of systemic conditioning of the “soil” by tumor-secreted factors.

## 9 EVs as diagnostic and prognostic biomarkers of metastasis

### 9.1 The potential of EVs in predicting and monitoring metastasis

**Figure 3:**
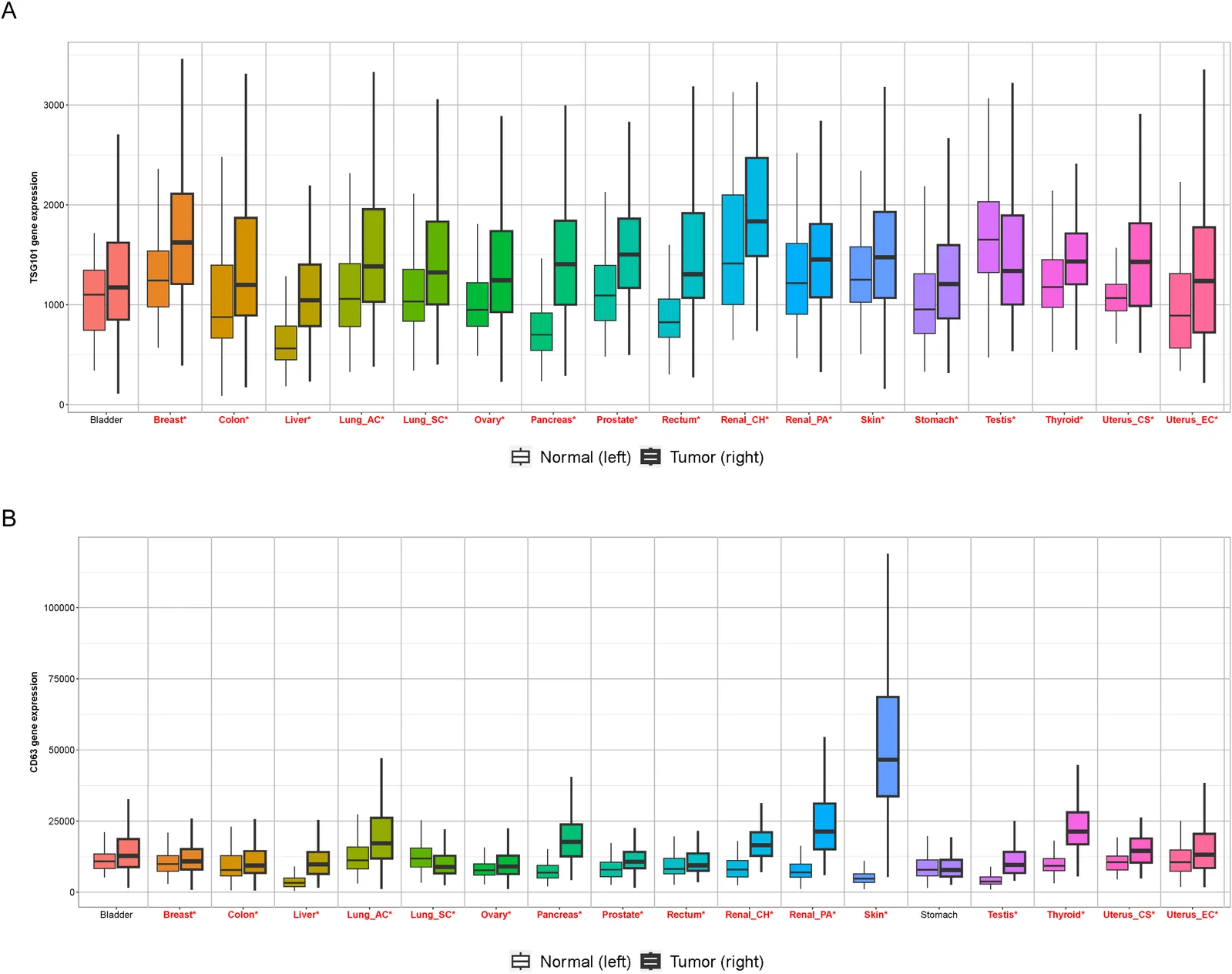
Expression of Exosomal markers in human cancers. The box plots show the mRNA expression levels of prominent exosome markers **(A)** *TSG101*, and **(B)** *CD63*, across paired tumor and normal tissue samples in various solid tumor types. Plots were created using the TNMplot.com (https://tnmplot.com/analysis/) platform. For each tumor type, the left box represents normal tissue, and the right box represents tumor tissue. This comparative analysis underscores the overexpression of classical exosome markers in tumor samples, supporting their utility as potential diagnostic and prognostic biomarkers. red*: Mann-Whitney p<0.05 and expression >10 in tumor or normal.

EVs contain a wealth of tumor-specific information, such as proteins, nucleic acids, and lipids, reflecting cancer cells’ molecular status (**Figure 3**). This makes them promising biomarkers for predicting and monitoring metastasis. As EVs can be easily isolated from body fluids (e.g., blood, urine, saliva), they offer a non-invasive approach to assess tumor progression and metastatic potential. For instance, specific miRNAs (e.g., miR-21, miR-23a) or proteins (e.g., TGF-β, integrins) in circulating EVs have been correlated with metastatic spread in cancers such as breast, lung, and CRC. Furthermore, EMT markers may be detected during EV profiling, indicating a shift towards a more invasive phenotype. Clinicians may be able to more precisely track the development of metastases, treatment response, and disease progression by monitoring alterations in the molecular makeup of EVs over time.

### 9.2 Advances in EV-based liquid biopsy for cancer patients

EV-based liquid biopsy holds promise for early cancer detection, identifying minimal residual disease, and tracking the emergence of drug resistance. Based on the molecular makeup of tumor-derived EVs, clinical research is currently investigating the potential of EVs for patient stratification, prognostic prediction, and therapeutic customization. However, challenges such as standardization of EV isolation methods and validation of specific EV biomarkers must be addressed to fully integrate EV-based liquid biopsy into routine clinical practice (123). Among others, a seminal report by Melo et al. has illustrated the importance of circulating exosomes in predicting pancreatic cancer prognosis (124).

EVs can be isolated from biofluids in a minimally invasive manner, providing real-time insights into the tumor’s molecular profile and its dynamic changes. Recent advances in technologies like next-generation sequencing (NGS), digital PCR, and high-resolution mass spectrometry have enhanced the sensitivity and specificity of EV-based assays for detecting cancer biomarkers.

## 10 Therapeutic implications

In therapeutics, engineered EVs offer innovative strategies for targeted drug delivery (125). Modified EVs can be loaded with chemotherapeutic agents, RNA therapeutics, or immunomodulatory molecules to enhance treatment efficacy while minimizing systemic toxicity (126, 127). Several clinical trials are evaluating the use of EVs as carriers for gene therapy and immunotherapy, showcasing their potential in personalized medicine (128). Despite these advancements, challenges such as standardizing EV isolation methods, ensuring reproducibility, and addressing off-target effects remain key hurdles in translating EV-based therapies into routine clinical practice.

Despite these challenges, several promising approaches are being explored, including EV inhibitors (e.g., GW4869, which blocks EV biogenesis), antibodies to block specific surface markers on EVs, and engineered EVs to deliver therapeutic payloads that suppress tumor progression.

## 11 Limitations and challenges in EV-based research and application

While EVs offer significant promise in understanding and managing metastatic progression, several limitations must be acknowledged. Firstly, the inconsistencies of following standardized protocols for EV isolation, characterization, and quantification lead to discrepancies across studies, complicating reproducibility and clinical translational efforts. Additionally, EV heterogeneity, arising from differences in size, biogenesis, cargo content, and cellular origin, makes it challenging to define specific functional subsets and their roles in metastasis.

Another key challenge is the uncertainty surrounding EV cargo loading mechanisms and organ-specific targeting. Although integrins have been implicated in directing EVs to specific organs, the full spectrum of molecular ‘address codes’ remains poorly understood. Furthermore, the functional transfer of EV cargo (e.g., miRNAs) to recipient cells, especially *in vivo*, is challenging to confirm definitively due to technical limitations in tracking cargo uptake and downstream gene regulation.

From a therapeutic standpoint, large-scale EV production, purification, and cargo loading present logistical hurdles. Moreover, issues such as ‘off-target effects’, ‘short circulating half life’, and ‘immunogenicity of engineered EVs’ must be resolved before clinical implementation can be fully realized. These limitations underscore the urgent need for advanced analytical tools, robust animal models, and integrative multi-omics approaches to delineate EV functions with greater precision and reliability.

## 12 Conclusion and future directions

Extracellular vesicles have revolutionized our understanding of intercellular communication in cancer metastasis. Their ability to transfer oncogenic signals, modulate the immune system, and establish pre-metastatic niches highlights their crucial role in disease progression. However, important ‘technical and biological limitations’, including EV heterogeneity, standardization challenges, and incomplete mechanistic understanding, must be addressed to unlock their full clinical potential.

Given their significance, future research should focus on refining EV-based liquid biopsy techniques for early cancer detection, standardizing isolation methods to improve reproducibility, and developing strategies to block pro-metastatic EVs while selectively enhancing anti-tumor EVs. Moreover, integrating multi-omics approaches with EV research can uncover novel biomarkers and therapeutic targets. Advancements in bioengineering can further optimize EV-based drug delivery systems for more precise and efficient cancer treatment. Bridging the gap between fundamental EV biology and clinical application will be essential in harnessing their full potential in oncology. As research progresses, EV-based diagnostics and therapeutics may pave the way for more effective, personalized interventions, ultimately improving patient outcomes in metastatic cancer.
