# Correction: Beyond boundaries: exploring the role of extracellular vesicles in organ-specific metastasis in solid tumors

**DOI:** 10.3389/fimmu.2026.2016288

**Published:** 2026-10-14

**Authors:** Goutham Nidhi, Vikas Yadav, Tejveer Singh, Deepika Sharma, Monica Bohot, Shakti Ranjan Satapathy

**Affiliations:** 1 Cell and Experimental Pathology, Department of Translational Medicine, Lund University, Skåne University Hospital, Malmö, Sweden; 2 Translational Oncology Laboratory, Department of Zoology, Hansraj College, University of Delhi, New Delhi, India

**Keywords:** extracellular vesicles, metastasis, EMT, cancer, exosome

Affiliation “Division of Cyclotron and Radiopharmaceutical Sciences, Institute of Nuclear Medicine and Allied Sciences (INMAS-DRDO), New Delhi, India” was erroneously included in the paper. This affiliation has now been removed for author “Tejveer Singh”.

The original version of this article has been updated.

